# Simultaneous Determination of Multiple microRNA Levels Utilizing Biotinylated Dideoxynucleotides and Mass Spectrometry

**DOI:** 10.1371/journal.pone.0153201

**Published:** 2016-07-05

**Authors:** Sobin Kim, Jungyun Park, Jeongkyeong Na, Gyoo Yeol Jung, Jungwook Hwang

**Affiliations:** 1 Graduate School for Biomedical Science & Engineering, Hanyang University, Seoul, Korea; 2 Department of Medical Genetics, College of Medicine, Hanyang University, Seoul, Korea; 3 School of Interdisciplinary Bioscience and Bioengineering, Pohang University of Science and Technology, Pohang, Gyeongbuk, Korea; 4 Department of Chemical Engineering, Pohang University of Sciences and Technology, Pohang, Gyeongbuk, Korea; Gustave Roussy, FRANCE

## Abstract

MicroRNAs (miRNAs) are important regulators of gene translation and have been suggested as potent biomarkers in various disease states. In this study, we established an efficient method for simultaneous determination of multiple miRNA levels, employing the previously developed SPC-SBE (solid phase capture-single base extension) approach and MALDI-TOF mass spectrometry (MS). In this approach, we first perform reverse transcription of miRNAs extracted using stem-loop primers. Then the cDNA is co-amplified with competitors, synthetic oligonucleotides whose sequences precisely match cDNA except for one base, and the amplicons serve as templates for a multiplexed SBE reaction. Extension products are isolated using SPC and quantitatively analyzed with MALDI-TOF MS to determine multiple miRNA levels. Here we demonstrated concurrent analysis of four miRNA levels utilizing the approach. Furthermore, we showed the presented method significantly facilitated MS analysis of peak area ratio owing to SPC. The SPC process allowed effective removal of irrelevant reaction components prior to MS and promoted MS sample purification. Data obtained in this study was verified with RT-qPCR and agreement was shown on one order of magnitude scale, suggesting the SPC-SBE and MS approach has strong potential as a viable tool for high throughput miRNA analysis.

## Introduction

MicroRNA (miRNA), a class of small (18-23nt) noncoding RNA, regulates translation of gene transcripts by typically suppressing the expression of target mRNAs [1,2]. Changes in the levels of miRNA have been implicated in onset and development of various diseases including cancer, renal diseases, diabetes, Alzheimer disease and cardiovascular diseases [3–7]. A large number of studies investigating roles of miRNA in the alteration of gene translation have been conducted to characterize disease states and to identify therapeutic pathways [8–10]. Most of these studies involve quantification of a group of miRNAs. Conventional gold standards of quantitative measurement of miRNA are RT-qPCR-based methods [11–14]. In these approaches, miRNAs are initially converted to cDNA and then each cDNA is amplified and quantified in real-time using fluorescence reading. They offer fast, accurate and sensitive analysis and have a wide range of applications. However, commonly one miRNA level is measured in one assay using these methods, which limits the throughput of miRNA analysis. A method that provides simultaneous evaluation of multiple miRNA levels as well as the speed and accuracy would significantly increase the throughput and efficiency of the assay. Therefore such a method is highly desirable and is in great demand. Here, we sought to develop a method to quantify multiple miRNA levels in one assay through an evolution of the approach we previously designed for multiplexed quantification of gene transcripts [15].

The method employs SPC-SBE (solid phase capture−single base extension) and MALDI-TOF MS (matrix-assisted laser desorption/ionization time-of-flight mass spectrometry) to ensure multiplexing capability, accuracy and speed [16–18]. MALDI-TOF MS is widely used in the analysis of large biological molecules such as oligonucleotides, peptides and proteins [17,19,20]. It is a highly accurate and fast method that is suitable for multiplexing, quantification and automation [21,22]. However, MALDI-TOF MS requires stringent sample purity. When irrelevant elements from preceding enzyme reactions, such as excess primers and salts, are not removed and introduced in MS with analytes, they can overlap with analyte peaks or produce adduct peaks, thus reducing accuracy in both qualitative and quantitative measurements. Therefore it is crucial to isolate analytes from salts and other reaction impurities prior to mass spectrometric analysis. SPC-SBE allows efficient sample purification for MALDI-TOF MS analysis through the use of biotinylated dideoxynucleotide triphosphates (biotin-ddNTPs) in primer extension reaction and subsequent isolation of extension products by a streptavidin-coated surface. Hence the SPC-SBE approach coupled with MALDI-TOF MS can produce convincing methods for oligonucleotide analysis. Previously we have used SPC-SBE and MALDI-TOF MS to develop a method for measuring gene transcript levels [15].

**Fig 1:**
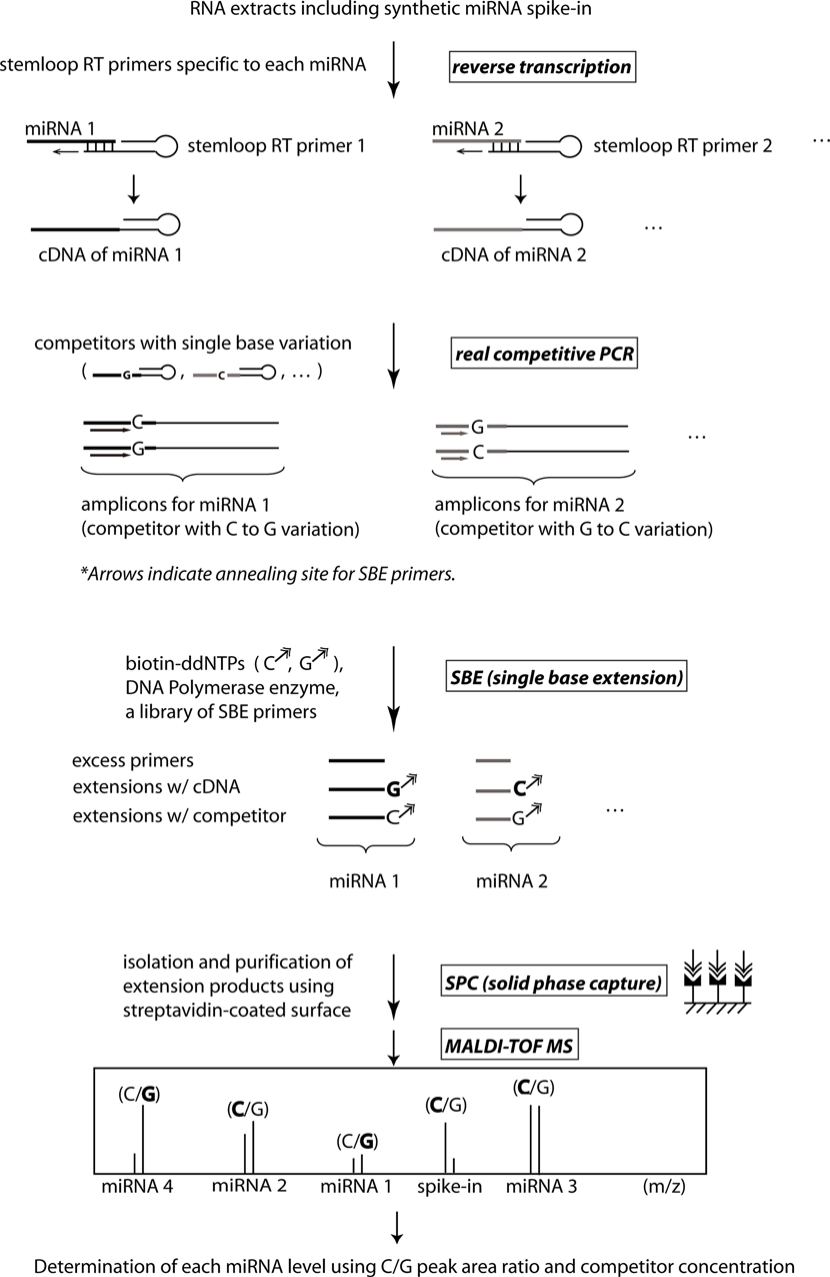
The SPC-SBE and MS approach for multiplexed miRNA quantification. Reverse transcription of miRNA using stem-loop primers is followed by co-amplification of cNDA and competitors with single base alterations. A library of SBE primers with distinct masses are extended by biotin-ddNTPs in a multiplexed SBE reactions. Two extension products are generated for each SBE primer, one for miRNA and one for its competitor. Then the extension products are purified in an SPC process and analyzed by MALDI-TOF MS. Area ratio of extension product peaks is used to determine the levels of miRNAs.

Here we present an approach that simultaneously determines multiple miRNA levels by implementing the previous method for quantifying gene transcripts. As shown in Fig 1, the approach engages stem-loop reverse transcription (RT) primers [23], cPCR (competitive PCR) [24], the SPC-SBE method and MALDI-TOF MS [16–18]. First, miRNA was reverse transcribed using a library of stem-loop RT primers. The stem-loop RT primers are designed to have six nucleotide(nt) overhangs that specifically anneal to the 3’ end of target miRNAs. Then cDNA is amplified in a multiplexed cPCR reaction with competitors of a known concentration. Competitors are synthetic oligonucleotide templates that have identical base sequences to the corresponding cDNA bar for one base alteration. Subsequently amplicons of the multiplex cPCR reaction serve as templates in a multiplexed SBE reaction. A library of SBE primers with distinct masses anneal right next to base alteration sites on amplicons of the competitors and cDNA, and SBE reactions are carried out using biotin-ddNTPs in a single reaction tube. Since the extension products carry biotin moieties, they can be captured on a streptavidin-immobilized solid surface, washed and released for MALDI-TOF MS. From the distinctive mass of each extension product, peaks are identified for specific miRNA and the corresponding competitor. The area under each peak is also measured to determine peak area ratios which are then used to decide the template ratios between miRNA and the competitor. Finally miRNA levels are calculated from the template ratios and initial competitor concentrations.

In this approach the SPC-SBE process eliminates excess primers and other components of enzyme reactions. This capability to isolate pure DNA extension fragments greatly supports multiplexed analysis and increases the quantification accuracy of MS analysis. The method quantifies miRNAs with accuracy and speed comparable to that of RT-qPCR based methods, but in a multiplexed way, significantly facilitating miRNA assays. Here we demonstrate concurrent measurement of the levels of four miRNAs utilizing the method in a proof-of-concept study.

## Materials and Methods

### Reverse transcription and competitive PCR

To demonstrate the method for multiplexed miRNA quantification, three miRNAs (miR-31, 24-3p and 142-3p) expressed in A549 human lung carcinoma cells were chosen. RNA was extracted using TRIzol (Invitrogen) following the manufacturer’s protocol. Prior to RNA extraction, a synthetic *Caenorhabditis elegans* miRNA mimic, cel-miR-48 (Bioneer), was added to the cell lysate as the spike-in control. RT reaction was performed in two steps. First, 1 μL of RNA extracts (~700 ng) containing cel-miR-48 were mixed with 0.1 μL of 10 mM dNTPs and deionized water, and heated at 65°C for 5 min followed by incubation on ice for 2 min. Subsequently, 4 μL of 5X RT buffer, 0.1 μL of Ribosafe RNase inhibitor (Bioline), 0.25 μL of Reverse Transcriptase (Thermo Scientific) and RT primers (0.25 μL of 4 μM each) for miR-31, 24-3p, 142-3p and the spike-in were added to the reaction mixture of 20 μL final volume. RT primers carried a stem-loop secondary structure and a short (6 nt) overhang at the 3’ end whose sequence matches the end of target miRNA as shown in Table 1. This final reaction mixture was then subjected to the following thermal cycling reaction: 16°C for 30 min, 60 cycles of 30°C for 30 sec, 42°C for 30 sec and 50°C for 1 sec, followed by 85°C for 5 min of heat-inactivation.

**Table 1:** Sequences of stem-loop RT primers (upper) and competitors (lower).

miRNA	Sequences of stem-loop RT primers*	Sequences of competitors**
miR-31	5’-GTCGATCCATGCAGGGTCCGAGGTATTCGCACTGGATACGAC**AGCTAT**-3’	5’-GTCGTATCCAGTGCAGGGTCCGAGGTATTCGCACTGGATACGACAGCTATGC**G**AGCATCTTGCCT-3’
miR-24-3p	5’-GTCGATCCATGCAGGGTCCGAGGTATTCGCACTGGATACGAC**CTGTTC**-3’	5’-GTCGTATCCAGTGCAGGGTCCGAGGTATTCGCACTGGATACGACCTGTTC**G**TGCTGAACTGAGCCA-3’
miR-142-3p	5’-GTCGATCCATGCAGGGTCCGAGGTATTCGCACTGGATACGAC**TCCATA**-3’	5’-GTCGTATCCAGTGCAGGGTCCGAGGTATTCGCACTGGATACGACTCCATAAA**C**TAGGAAACACTACA-3’
Spike-in	5’-GTCGATCCATGCAGGGTCCGAGGTATTCGCACTGGATACGAC**TCGCAT**-3’	5’-GTCGTATCCAGTGCAGGGTCCGAGGTATTCGCACTGGATACGACTCGCAT**G**TACTGAGCCTACCTCA-3’

*Sequences specific to miRNA are shown in bold.

**Sequence variations are shown in bold and underlined.

**Fig 2:**
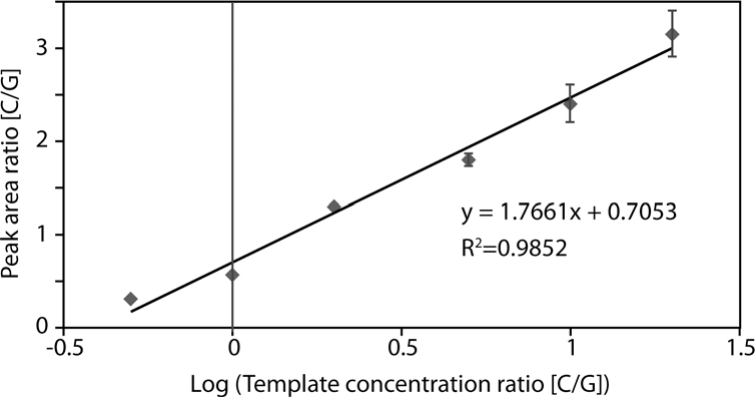
Calibration curve established to calculate the initial miRNA concentration in the competitive PCR reaction.

For cPCR, four competitor templates were co-amplified with cDNA. Competitors were synthetic DNA oligonucleotides about 65~67 nt long and had base sequences precisely matching cDNA from miRNA except for one base (Table 1). Various competitor concentrations in the range of 0.1 to 10 fmol (equivalent to 0.01–1 pM) were tested at varied ratios between the two biotin-ddNTPs in SBE reaction, for each miRNA. In the presented method, the fraction of mass spectral peak area and thus the accurate quantification by mass spectrometry was significantly affected by the biotin-ddNTP ratio, as well as the competitor concentration. We chose a competitor concentration that produced a peak area ratio within the range covered by the standard curve shown in Fig 2, which would ensure higher accuracy for quantitative mass spectrometry.

Reaction mixtures for cPCR contained the following: 20 pmol each of four forward primers and 80 pmol of reverse primer common to all; 10 μL of cDNA from the reverse transcription step above; competitor templates (4 × 10^−6^ μM for miR-31, 10^−4^ μM for miR-24-3p, 10^−4^ μM for miR-142-3p, and 4 × 10^−6^ μM for the spike-in), 20 μL of 5X PCR buffer, 14 μL of 1 mM dNTPs, 2 units GoTaq polymerase (Promega), and deionized water to make the final volume of 100 μL. Sequences of forward primers were 5’-GCCGAAGGCAAGATGCT-3’ (miR-31), 5’-GCCGATGGCTCAGTTCAG-3’ (miR-24-3p), 5’-GCCGATGTAGTGTTTCCTA-3’ (miR-142-3p), and 5’-CGATGAGGTAGGCTCAGTA-3’ (spike-in). The reverse primer sequence was 5’-GTGCAGGGTCCGAGGT-3’. All competitors and primers were commercially purchased (IDT).

Thermal cycle reactions were performed in a BIO-RAD T100™ thermal cycler: an activation stage of 94°C for 3 min followed by incubation at 40°C for 1 min; an amplification stage of 94°C for 15 sec, 50.5°C for 30 sec, and 72°C for 15 sec for 35 cycles; and a final extension stage of 72°C for 5 min. Products of cPCR reaction were then cleaned up with 5 units each of Shrimp Alkaline Phosphatase and Exonuclease I (New England Biolabs), at 37°C for 90 min. The enzymes were then heat-inactivated at 94°C for 15 min.

### SPC-SBE

SBE reaction mixtures contained 15 μL of clean-up reaction product, 125 pmol each of biotin-11-ddCTP (Jena Biosciences) and biotin-11-ddGTP (Jena Biosciences), 2 units ThermoPol DNA Polymerase (New England Biolabs), 6 μL of reaction buffer, four SBE primers (10 pmol for miR-31, 30 pmol for miR-24-3p, 40 pmol each for miR-142-3p and the spike-in), and water in a 60 μL volume. The SBE primer sequences are shown in Table 2. Reaction mixtures were then subjected to the following thermal cycle: 94°C for 3 min, 30 cycles of 94°C for 10 sec, 52°C for 30 sec and 44°C for 20 sec.

**Table 2:** SBE (Single Base Extension) primers.

			Mass of SBE products (Da)*	
miRNA	Sequences	Mass (Da)	Biotin-ddC	Biotin-ddG
miR-31	5’-AGGCAAGATGCT-3’	3694	4359	**4398**
miR-24-3p	5’-ATGGCTCAGTTCAGCA-3’	4881	5546	**5585**
miR-142-3p	5’-CCGATGTAGTGTTTCCTA-3’	5481	**6146**	6185
Spike-in	5’-CGATGAGGTAGGCTCAGTA-3’	5893	6558	**6597**

*Extension products with miRNAs and competitors are shown in bold and regular, respectively.

Extended SBE primers were separated from other reaction components using streptavidin-coated magnetic beads (New England Biolabs). Briefly, 125 μL of bead solution were pre-washed twice with 125 μL of binding/washing (B/W) buffer (1 M ammonium chloride, 2X Tris-EDTA) and re-suspended in 125 μL B/W buffer. SBE products were mixed with the bead solution and allowed to incubate at room temperature for 30 min under occasional mild mixing. The bead solution was then washed twice with 200 μL B/W buffer and twice with 200 μL deionized water, followed by re-suspension in 10 μL of deionized water. Bound DNA extension products were eluted from the streptavidin-coated beads by heating the bead solution at 75°C for 5 min. Isolated extension products were then further desalted using a ZipTip™ C18 column (Millipore) to ensure high accuracy for quantitative MS analysis.

### MALDI-TOF MS

Isolated SBE products were analyzed using the Axima Confidence (Shimadzu Biotech) MALDI-TOF MS instrument in linear negative mode. First, they are dissolved in 1 μL of deionized water, mixed with 1 μL matrix solution, and hand-spotted on an MS sample plate to air-dry. Matrix was composed of 27 mg of 3-hydroxypicolinic acid (Fluka) and 5 mg of ammonium citrate (Fluka) dissolved in 500 μL of 50% acetonitrile solution (Sigma). MS measurement parameters were set to 92~97 unit laser intensity with the ion gate off at the ion chamber voltage 20kV and pulsed extraction at 5700 Da. An accumulated spectrum was generated by measuring five areas of a sample spot with 30 laser shots taken for each area, on average.

### MS calibration for quantification

As an external calibration, we established a standard curve using a couple of synthetic DNA templates that are identical in sequence except for one base (one carrying G and the other C) in the middle, mimicking the cDNA/competitor pair. The sequence of the synthetic templates was 5’- GTCGTATCCAGTGCAGGGTCCGAGGTATTCGCACTGGATACGACCTGTTC **C** TGCTGAACTGAGCCA -3’ and 5’- GTCGTATCCAGTGCAGGGTCCGAGGTATTCGCA CTGGATACGACCTGTTC **G** TGCTGAACTGAGCCA -3’. The SBE primer sequence was 5’-ATGGCTCAGTTCAGCA -3’ (Mw 4881). The synthetic templates mixed at various ratios (C/G) of 0.5, 1, 2, 5, 10 and 20 were co-amplified in one reaction tube. Amplicons were used to extend an SBE primer at the sequence variation site with biotin-ddCTP and biotin-ddGTP in an SBE reaction. After isolation by streptavidin immobilized surface, the C- and G-extension products of SBE reaction (5546 Da and 5585 Da, respectively) were analyzed with MALDI-TOF MS. The peak areas of C and G extension products were measured to calculate the area ratio which was then plotted to the initial synthetic template ratio. All experiments were performed in triplicates and data from three sets of experiments ware combined.

### RT-qPCR

RT-qPCR was carried out on a BIO-RAD CFX Connect™ real time PCR cycler using a Qiagen QuantiTect® SYBR® Green PCR kit. Each reaction contained 2.5 μL of SYBR Green master mix, 10 pmol each of both forward and reverse PCR primers (sequences shown above), 1 μL of cDNA from the reverse transcription step above, and deionized water for a final reaction volume of 10 μL. Thermal cycling conditions included 50°C for 2 min; 95°C for 10 min; 40°C for 1 min; 35 cycles of 95°C for 15 sec and 50°C for 1 min; and a melting curve analysis stage raising the temperature from 50°C to 99°C at a rate of 0.1°C/sec.

## Results

### Calibration for quantitative mass spectrometry

For quantitative MS analysis of miRNA levels, we established an external calibration curve using two synthetic oligonucleotide templates (Fig 2). Both C- and G- extension peaks with good signal intensity were observed for all template ratios tested (C/G ratio of 0.5, 1, 2, 5, 10 and 20). Peak area ratio (C/G) was then measured and plotted to the corresponding template ratio on a semi-Log scale, as shown in Fig 2. The error for each data point ranged between 1% and 21% and the R^2^ value was 0.9852. In the tested range, template ratio was not linearly proportional to the MS peak area ratio. This could be due to the difference in the extension reaction rates of biotin-ddCTP and biotin-ddGTP or in ionization efficiencies during MS analysis between C and G extension products.

### Multiplex determination of miRNA levels

**Fig 3:**
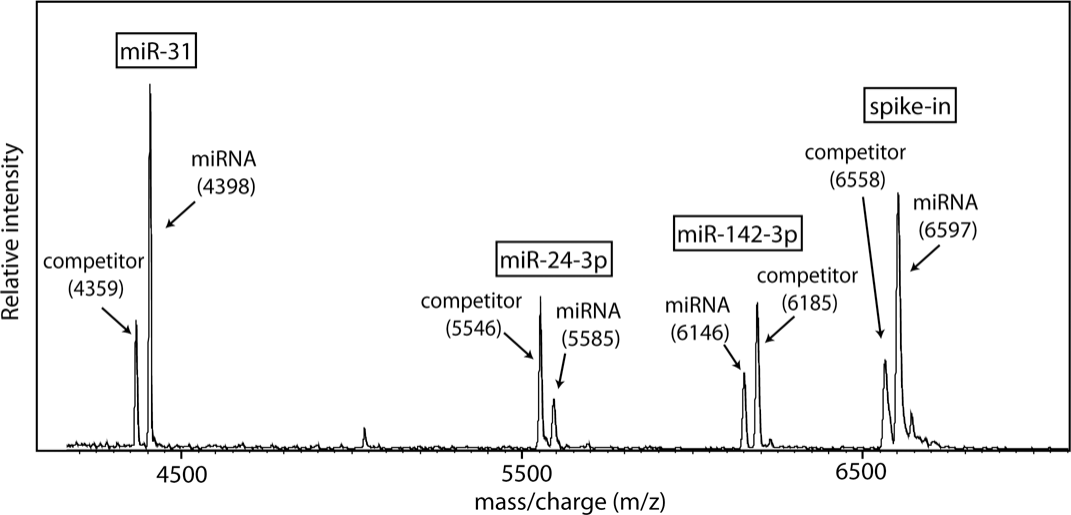
Mass spectrum for multiplexed analysis of four miRNA levels. Levels of cellular and spiked miRNA are determined using the peak area ratio and the initial concentration of the corresponding competitors.

We performed multiplex cPCR using cDNA of four miRNAs (miR-31, 24-3p, 142-3p and spike-in) and their competitors. Products of cPCR were subjected to the SPC-SBE process followed by MALDI-TOF MS of the extension products. A representative mass spectrum for multiplex determination of miRNA levels using the SPC-SBE and MS approach is shown in Fig 3. Eight completely resolved peaks (four doublet peaks) were produced for the four miRNAs. All peaks were correctly identified as an miRNA product or a corresponding competitor using the mass-per-charge ratios. For example, the first peak at mass of 4359 Da and the second one at 4398 Da represent the C extension product (competitor) and the G extension product of the miR-31 SBE primer, respectively. In the mass spectrum, only SBE extension product peaks were observed and a few salt adduct peaks appeared with minimal intensity. This indicates other irrelevant reaction components such as SBE primers were successfully eliminated by the SPC process.

To quantify each miRNA, we measured the C/G peak area ratio from a doublet peak and used the calibration curve shown in Fig 2 to decide a concentration ratio between miRNAs and competitors (Table 3). Salt adduct peaks that did not constantly appear had a peak area that generally was too small to make any significant difference in the concentration ratio. Thus we have considered only the analyte peak areas for the calculation. As shown in Table 3, the concentration ratio was then converted to the initial level of miRNA in cPCR reaction using the concentration of a corresponding competitor added to the cPCR reaction. Since we optimized the amount of competitors as explained in the Materials and Method, the miRNA and the corresponding competitor peaks had comparable areas under the peak: the peak area ratio (C/G) was found in the range between 0.37 and 2.4 (Table 3). The concentration ratio calculated using the calibration curve ranged between 0.62 and 251. It was revealed that miR-142-3p had the highest level of expression while the spike-in showed the lowest level (Table 3).

**Table 3:** Peak area ratios measured from MS, calculated concentrations of miRNAs in cPCR reaction, and relative levels of miRNA measured in RT-qPCR.

miRNA	Peak area ratio* (C/G)	Initial conc. of competitors (10^−6^ μM)	Initial conc. of miRNAs** (10^−6^ μM)	Fold difference from conc. of spike-in	Levels of miRNA relative to spike-in, measured by RT-qPCR*** (difference from the MS-method)
miR-31	0.37±0.02	4	6.19±0.12	1.07±0.03	2.85±0.21 (166%)
miR-24-3p	2.4±0.2	100	11.0±3.26	1.89±0.55	2.51±0.14 (33%)
miR-142-3p	0.56±0.03	100	121±5.12	20.9±0.80	25.7±3.55 (23%)
Spike-in	0.42±0.01	4	58.0±0.05	1	1

* Peak areas were measured from an accumulated spectrum and the data were obtained from four sets of experiments, with each set in duplicate. Peak area ratios for the four sets of experiment were 0.37, 0.34, 0.41, 0.36 (miR-31); 2.9, 2.3, 2.5, 1.9 (miR-24-3p); 0.52, 0.58, 0.64, 0.49 (miR-142-3p); 0.41, 0.44, 0.42, 0.41 (spike-in).

** The initial concentrations of miRNAs were calculated using the calibration curve shown in Fig 2.

*** Two sets of RT-qPCR were performed for the four miRNAs, each reaction in duplicate. Relative miRNA levels were determined using Cq values. Cq values were 14.49, 14.84 (miR-31); 14.71, 14.98 (miR-24-3p); 11.69, 11.30 (miR-142-3p); 16.18, 16.18 (spike-in).

The presented method allowed us to calculate the concentration of spike-in control as well as miRNAs as shown in Table 3. Since the amount of spike-in added to the sample is known (2 fmol per μL final extract), we could compare it with the calculated value shown in Table 3, as an additional guideline to gauge the quantification accuracy of the method. The initial concentration of the spike-in was calculated to be 5.8×10^−6^ μM (Table 3), from which the total amount of the spike-in control in the cPCR reaction (total volume of 100 μL) is determined as 0.58 fmol. Then we can estimate that the amount of spike-in in the RNA extract was 1.16 fmol per μL final extract at most, considering the volume change in reaction steps followed. This estimated value is within an order of magnitude from the actual amount of spike-in control added to the sample. The result appeals the presented method has high feasibility for accurate quantitative analysis of miRNA levels.

### Verification by RT-qPCR

The results obtained by the SPC-SBE and MS approach were confirmed using RT-qPCR. RT-qPCR reaction was performed for the four miRNAs, each reaction in duplicate, and the threshold cycle (Cq) values were measured 14.67±0.11, 14.85±0.08, 11.50±0.23 and 16.18±0.01 for miR-31, miR-24-3p, miR-142-3p and cel-miR-48, respectively. Then we calculated level of each miRNA with respect to spike-in using the difference in Cq values. It was assumed the PCR efficiency was optimal as the amplicons were short (less than 100 nt) and that each increase of one cycle corresponded to a two-fold decrease in miRNA levels.

As shown in Table 3, both quantification methods verified that the level of miR-142-3p in the cPCR reaction was at least twenty times higher than the other miRNAs. The relative expression levels of miR-31, 24-3p and 142-3p measured by RT-qPCR were higher than the values determined by the SPC-SBE based method by 166%, 33% and 23%, respectively. However, data obtained from the two quantification methods agreed on the one order of magnitude scale, which strongly supports the viability of MS-based miRNA quantification.

## Discussion

We have demonstrated a method to simultaneously determine four miRNA levels (including one synthetic miRNA as a spike-in control) utilizing SPC-SBE, cPCR and MALDI-TOF MS. The method employs SPC, a molecular affinity separation process, to allow the isolation of SBE reaction products from other irrelevant reaction elements prior to MS analysis [16]. This significantly facilitates MALDI-TOF MS because irrelevant reaction elements can complicate mass spectrum and lower the accuracy of MS analysis, when measured together with extension products. In particular, quantitative MS measurements can be stalled by irrelevant peaks and salt adduct peaks that overlap with the analyte peaks. The SPC process can effectively eliminate them and thus provides a critical aid in quantification by MALDI-TOF MS. Furthermore, enhanced MS sample purity by the SPC process helps co-crystallization of the matrix-analyte mixture resulting in an improved signal-to-noise ratio. The presented method utilizing SPC produced mass spectra of eight extension products, as shown in Fig 3. All four doublet peaks had good signal intensity and adducts or primer peaks were absent or negligible. Thus determination of peak area was simple and fast, leading to efficient quantification of miRNA by MALDI-TOF MS.

The SPC-SBE and MS approach can also provide higher levels of multiplexing, thus the throughput, in miRNA analysis. MALDI-TOF MS is restricted in mass range for optimal measurement of oligonucleotide analytes and the number of peaks that can be included in this mass range is limited. Therefore, when analyzed without irrelevant reaction components, more extension products can be measured in one mass spectrum increasing the level of multiplexing. The SPC-SBE approach currently allows up to fifty-fold multiplexing, which is at the highest level of multiplexing among MS-based methods to the best of our knowledge. The SPC-SBE and MS approach for miRNA analysis allows simultaneous detection of four miRNA levels as shown in this study and the multiplexing level of the approach could be readily increased with minimal modification. Moreover, multiplexed analysis of miRNAs that carry highly similar sequences (i.e. isomeric miRNAs) could be feasible with careful design of competitors and SBE primers, and use of tandem mass spectrometry.

In addition, the SPC-SBE and MS approach generates two extension products with an identical length and an adequate mass difference, by utilizing biotin-ddNTPs in the SBE reaction. In this study, we chose all competitor sequences to ensure that single base variation from the corresponding miRNA sequence was either G to C or C to G. The resulting extension products had a mass difference of 39 Da warranting the full resolution of extension peaks, while the peak intensity declining, which is typically observed as analyte mass increases, would not be significant with this mass difference. While other variations such as G/T (mass difference of 50 Da), A/T (mass difference of 56 Da), and C/T (mass difference of 89 Da) can be also considered as they have been shown to produce well-resolved mass peaks [16,18], we believe that keeping all variations in the form of C/G is feasible for majority of applications and most desirable for the simplicity of the method.

RT-qPCR verification of the results revealed that miRNA levels determined by the two quantification methods were on the same order of magnitude and the largest discrepancy between two was a roughly three-fold difference in the relative expression level of miR-31 (1.07 by the presented approach and 2.85 by RT-qPCR, Table 3). As outlined in Results, we have observed about 21% variation from the mean with a relatively high R^2^ value in the calibration curve for MS quantification. Therefore, while the overall accuracy of the SPC-SBE and MS method could still be lower than indicated by the error value for MS quantification shown in Table 3, the error in RT-qPCR measurement can also account for the deviation in miRNA levels measured by the two methods. Causes of the errors introduced in quantification by MALDI-TOF MS could include heterogeneity of matrix-sample crystals and variable ionization efficiency. Automated systems that can more accurately handle small volumes of sample solutions and produce smaller sample spots for MS would significantly increase repeatability and accuracy of peak area measurement, lowering overall errors in quantification by MALDI-TOF MS.

In this study, we established a multiplexed assay for quantification of miRNAs employing SPC-SBE, cPCR and MALDI-TOF MS. The high capacity of multiplexing and the rapid and accurate quantification provided by the SPC-SBE approach and MALDI-TOF MS suggest strong possibility that this method makes a robust tool for miRNA analysis. With future improvement in MS analysis such as discovery of new matrices for better analyte ionization and automated sample handling system as stated above, the presented method will be further improved for quantification accuracy, reproducibility and throughput, and could provide a potent platform for high throughput miRNA assay in clinical and commercial settings.

## Abbreviations

SPC-SBE: solid phase capture−single base extension
MALDI-TOF MS: matrix-assisted laser desorption/ionization time-of-flight mass spectrometry
cPCR: competitive PCR
